# Comparison of short-term and long-term renal function effects of vasopressin and norepinephrine in patients with septic shock: a systematic review and meta-analysis

**DOI:** 10.3389/fphar.2025.1669636

**Published:** 2025-11-18

**Authors:** Hongyu Wang, Xilin Liu, Hong Zhang

**Affiliations:** School of Nursing, Guangdong Medical University, Dongguan, Guangdong, China

**Keywords:** vasopressin, renal function, norepinephrine, septic shock, meta-analysis

## Abstract

**Background:**

Vasopressin and its derivatives, as second-line vasoactive agents, are increasingly being applied in the treatment of septic shock, but their effects on major organs, particularly the renal system, remain inadequately evaluated.

**Methods:**

A systematic search was conducted based on 4 online databases Embase, PubMed, ScienceDirect, and Scopus, for studies published up to April 2025 that compared the renal function outcomes between vasopressin versus norepinephrine. All the studies enrolled adult patients with septic shock. Both short-term outcomes (urine output, serum creatinine levels) and long-term outcomes (acute kidney injury (AKI) rate, renal failure (RF), days free of RF, and renal replacement therapy (RRT)-use rate) were assessed.

**Results:**

A total of 13 studies met the inclusion criteria, comprising 10 RCTs and 3 retrospective cohort studies, with a total of 2,024 septic shock patients (aged 46.76–68 years) engaged. Meta-analysis showed no significant differences between the vasopressin and norepinephrine groups in the incidence of AKI (Risk Ratio (RR) = 1.07, 95% CI [0.86, 1.33], P = 0.53), days free of RF (MD = 1.52, 95% CI [−2.21, 5.25], P = 0.43), incidence of RF (RR = 1.01, 95% CI [0.85, 1.19], P = 0.94), or urine output (MD = −161.93 mL, 95% CI [−690.31, 366.45], P = 0.55). However, vasopressin was associated with a significantly lower serum creatinine level (MD = −0.15 mg/dL, 95% CI [−0.29, −0.02], P = 0.028) and a reduced RRT utilization rate (RR = 0.76, 95% CI [0.62, 0.93], P < 0.01) compared to norepinephrine.

**Conclusion:**

Vasopressin demonstrates potential renal protective effects in the management of septic shock, as evidenced by a significant reduction in serum creatinine levels and a decreased need for renal replacement therapy compared with norepinephrine. However, the evidence supporting its benefit in reducing the incidence of AKI and RF, or prolonging the days free of RF remains of low quality.

## 1 Introduction

Septic shock represents the severe progressive stage of sepsis and is characterized by systemic vasodilation, increased vascular permeability, hypovolemia, and myocardial dysfunction (Angus and van der Poll, 2013). It is strongly associated with high mortality rates. A large-scale epidemiological study (Bauer et al., 2020) reported a pooled 30-day in-hospital mortality for septic shock in Europe and the United States of 34.7% (95% CI 32.6%–36.9%). The cornerstone of treatment for this disease is restoring tissue and organ perfusion through fluid resuscitation and maintaining a mean arterial pressure (MAP) of at least 65 mmHg. When adequate MAP cannot be achieved with fluid resuscitation, vasopressors are recommended to get in and help correct tissue hypoxia (Gavelli et al., 2021).

Current guidelines recommend norepinephrine (NE) as the first-line vasoactive agent, followed by dopamine (DA). The molecular mechanism of these agents primarily involve activation of α1- and α2- adrenergic receptors on vascular smooth muscle cells, leading to vasoconstriction, thereby increasing peripheral vascular resistance, raising MAP, and improving organ perfusion (Rhodes et al., 2017). However, high doses of these catecholamines are associated with serious adverse effects, including immunosuppression, metabolic disturbances, and extensive myocardial cell death (Cattaneo et al., 2023). Furthermore, during septic states, some patients develop vascular resistance to catecholamines, rendering even high doses of NE and DA ineffective in correcting hypotension (Gordon et al., 2012). In such cases, the second-line vasopressor arginine vasopressin (AVP) has gained increasing attention. Evidence suggests that low-dose AVP can stabilize hemodynamics and enhance tissue perfusion in patients with septic shock just as NE can do. Moreover, it can be used in combination with NE or DA to reduce the required doses of these agents, highlighting its potential therapeutic value in septic shock management (Andersen et al., 2023).

Arginine vasopressin (AVP) and its analogues are being increasingly utilized, particularly in cases of catecholamine-resistant or refractory shock. However, as their use becomes more widespread, associated adverse effects are also being increasingly recognized. A meta-analysis conducted by Nagendran et al. (2019) which encompasses a total of 1,453 patients, compared the efficacy of AVP and NE and found AVP was associated with a higher incidence of cerebral ischemia and a lower incidence of arrhythmia-related adverse events. Additionally, the study suggested a potential reduction in renal replacement therapy (RRT) usage with AVP, while the quality of evidence was low.

The inflammatory factors released by sepsis itself, such as TNF - α and IL-1, can activate the oxidative stress pathway and injury renal tubular cells; NE helps maintain effective perfusion pressure, thereby improving glomerular filtration rate, mitigating tubular cell damage and reducing the risk of acute kidney injury (AKI) (Al-Husinat et al., 2023). However, animal study have indicated that the strong vasoconstrictive effects of NE on both afferent and efferent glomerular arterioles may reduce glomerular filtration rate, creatinine clearance, and urine output, thereby increasing the risk of AKI (Anderson et al., 1981). In contrast, AVP primarily constricts efferent arterioles and has minimal impact on afferent arterioles, thereby increasing renal perfusion pressure and enhancing renal blood flow, which contribute to improved renal function (Gessner, 2006). Nonetheless, these findings remain largely theoretical and require further clinical validation.

Given the ongoing debate and limited high-quality evidence, this study aims to systematically review the literature and synthesize data regarding renal outcomes associated with AVP and NE in septic shock, to provide more robust evidence for clinical decision-makers.

## 2 Materials and methods

### 2.1 Data source

We searched online databases Embase, Pubmed, Science Direct, Scopus for studies published up to April 2025, with the terms vasopressin, norepinephrine, septic shock, renal failure, acute kidney injury. According to different databases, the retrieval process was adjusted appropriately. We also searched for relevant literature in Google Scholar and Clinicaltrials.org for additional studies.

### 2.2 Eligibility criteria

The eligibility criteria was pre-defined before screening. All included studies must be RCTs or cohort studies. Meeting records, surveys, reviews, evidence summaries, and letters that do not provide data were excluded. The subjects of the study must be adult patients (aged >18 years) with diagnosed septic shock, with clear diagnostic criteria such as MAP below 65 mmHg. Studies involving other types of shock other than septic shock (e.g., neurogenic shock, anatomical Shock) or animal models were excluded. The studies were required to have at least two intervention groups (vasopressin, norepinephrine). Studies with small sample sizes (n < 10) in each group will be excluded. Studies were required to report outcomes related to kidney function, such as the incidence of AKI, RF, and the rate of renal replacement therapy (RRT)-use. Studies that do not assess renal function outcomes or fail to provide available data were excluded.

### 2.3 Study screening

After the literature search was done, two reviewers read the titles and abstracts of the literature to eliminate duplicate references, which was defined as literature with similar titles, authors, publication years, and experimental content. Next, based on the pre-defined criteria, the two reviewers screen the titles and abstracts to preliminarily determined the included studies. Full text of relevant literature and its ancillary data were retrieved from the online databases. Studies without a full text or available data would be excluded.

Subsequently, a detailed full-text review was performed by the two reviewers to determine the final included studies. In cases of disagreement regarding study eligibility, inclusion or exclusion was decided through discussion until a consensus was reached.

### 2.4 Quality and bias assessment

The Cochrane Risk of Bias V2.0 (Sterne et al., 2019) was provided to assess the bias with three levels, “low,” “some concern of risk,” or “high.” Given that the cohort studies are not RCTs, they were assessed with appropriate adjustments. The item “randomization process” was rated as high risk, while the item “Deviation of intended intervention” was rated as “some concern of risk.”

### 2.5 Outcomes

The outcome measures are divided into short-term renal function outcomes and long-term renal function outcomes. The short-term outcomes were serum creatinine levels, creatinine clearance, and urine output within 24–48 h. The long-term outcomes were incidence of AKI, incidence of renal failure, days free of RF, and RRT use rate.

### 2.6 Data extraction

Two researchers independently reviewed the full text and the data of the included studies, structurally extracted data such as author, publication year and month, number of participant cases, participant age, gender ratio, intervention dose. The data were cross-checked for consistency. After being extracted, the data were standardized using consistent units. For example, serum creatinine levels reported in different units—such as mg/dL and μmol/L—were converted to a uniform unit (mg/dL), with values in μmol/L converted by dividing by 88.4.

### 2.7 Effect size and pooling

The differences in AKI incidence, renal failure incidence, and RRT use rate between the vasopressin and norepinephrine groups were reported using RR (Risk Ratio) and its 95% CI, while days free of RF, serum creatinine levels, creatinine clearance, and urine output were reported using Mean Difference (MD) and 95% CI. The above effect quantities were pooled using the “meta” or “metafor” package in R language. If there is statistically significant heterogeneity between literature, the random effects model’s Dersimonian Laird method is used for calculation; On the contrary, the Mantel Haenszel (MH) method with fixed effects model is used for calculation.

### 2.8 Heterogeneity detection

Cochrane Q statistic was used to detect whether there is heterogeneity between literature. P < 0.05 indicates the presence of significant heterogeneity, otherwise there is no heterogeneity.

### 2.9 Subgroup analysis

Subgroup analyses were performed to explore potential sources of heterogeneity in outcomes that demonstrated significant variability during the analysis.

### 2.10 Influence analysis

The “Labbe” function provided by the “metafor” package was used to plot a L'Abbé plot (L'Abbé et al., 1987), and the “qqnorm” function was used to plot a normal quantile-quantile (QQ) plots (Shi et al., 2017) to demonstrate the concentration of literature.

### 2.11 Publication bias

The “funnel” function of the “meta” package was used to perform publication bias analysis on the effect sizes of the outcomes, presented in a funnel plot.

### 2.12 Statistical analysis

Data statistics were completed under R language (v4.4.1) environment that was integrated in Rstudio (v764). P < 0.05 was considered statistically significant.

### 2.13 PROSPERO registration

The protocol for this systematic review and meta-analysis was prospectively registered with the International Prospective Register of Systematic Reviews (PROSPERO) on [Date of Registration] under registration number CRD420251105774.

## 3 Results

### 3.1 Study selection

**FIGURE 1:**
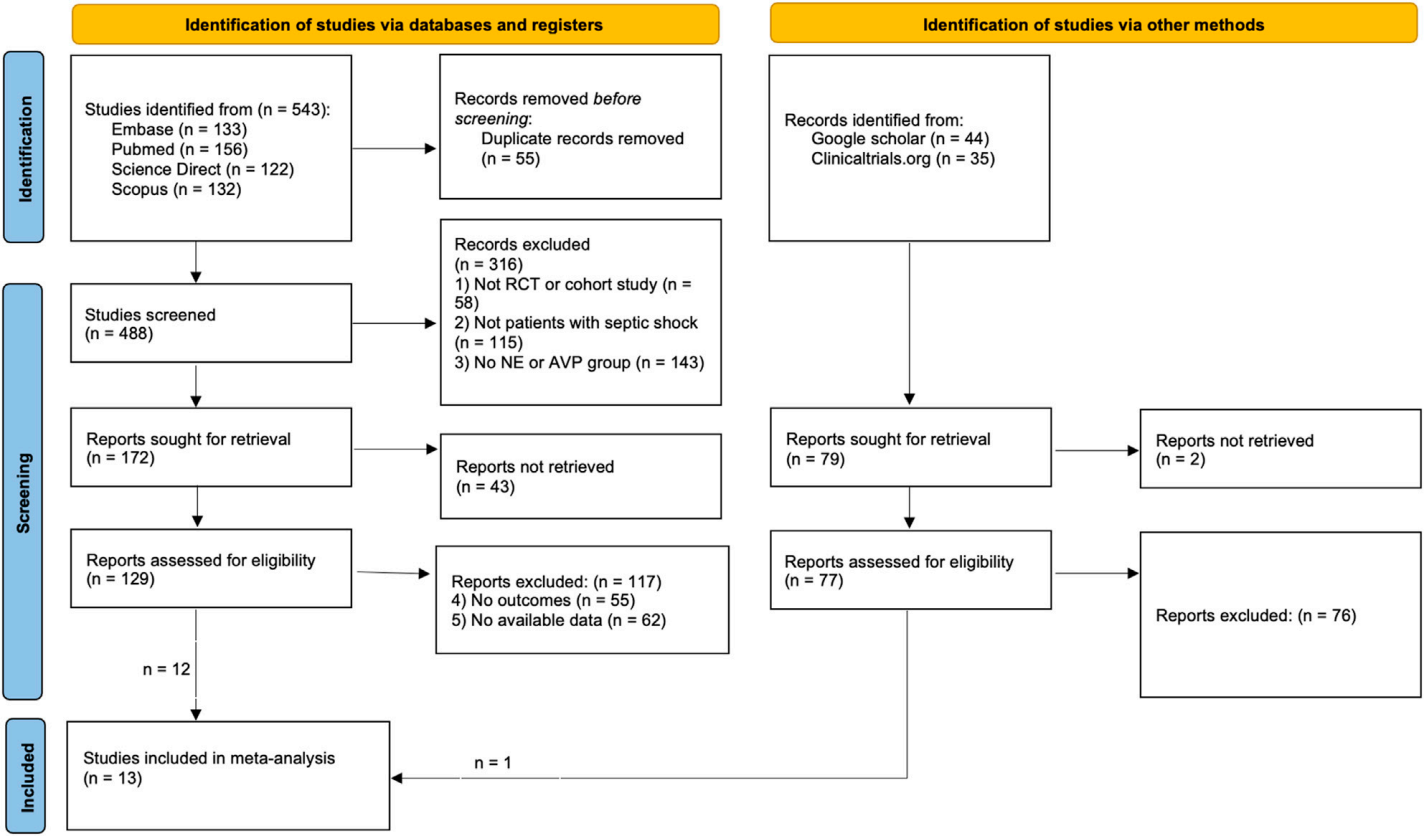
The flow chart.

Figure 1 shows the literature selection process flowchart. The search strategy initially identified 543 articles, of which 55 were duplicates and were excluded. After preliminary screening, a total of 316 articles were disqualified and excluded. The remaining 172 articles entered the fine screening process, of which 43 articles could not obtain the full text. After careful reading and further screening, a total of 12 articles entered the quantitative analysis within the remaining 129 articles; We initially retrieved 44 articles from Google Scholar and 35 articles from Clinicaltrials.org, and after exclusion, we obtained 1 article for quantitative analysis. A total of 13 articles (Russell et al., 2008; Gordon et al., 2016; Hajjar et al., 2019; Mehta et al., 2013; Gordon et al., 2010; Hammond et al., 2019; Hammond et al., 2018; Morelli et al., 2009; Choudhury et al., 2017; Dünser et al., 2003; Patel et al., 2002; Lauzier et al., 2006; Hall et al., 2004) were included in the final analysis.

During the screening process, we excluded all studies with non-septic shock patients as research subjects. Although the study conducted by Cheng et al. (2018) also evaluated the effects of AVP and NE, the included patients were patients with vasoplegic shock, not septic shock, and were therefore excluded. The study by Lin et al. (2005) focused on rats and was therefore excluded. Klinzing et al.'s RCT (Klinzing et al., 2003) had a small number of cases in each group (n < 10) and was therefore excluded. Daley MJ et al.'s study (Daley et al., 2013) did not include renal function outcomes and was therefore excluded. Jeon K et al.'s study (Jeon et al., 2018) explored the possible effects of discontinuation on patients, rather than the effects of continuous infusion on patients, and therefore was excluded. We did not list all the studies that were initially considered but ultimately excluded, only 5 representative ones were listed (Table 1).

**TABLE 1:** Excluded literature and reasons (not all listed).

Authors and publication year	Reason for exclusion
Cheng et al. (2018)	Involving non-septic shock patients
Lin et al. (2005)	Involving animal models
Klinzing et al. (2003)	The sample size for grouping is too small
Daley et al. (2013)	No renal function outcomes
Jeon et al. (2018)	Inappropriate intervention process

### 3.2 Characteristics of the included studies

Thirteen qualified articles were identified through literature screening, including 2,040 patients with septic shock. These studies were published between 2002 and 2019. Among them, there were 10 RCTs and 3 retrospective cohort studies. The median age range of patients is 46.76–68 years old. Gordon et al. (2016) can be divided based on whether hydrocortisone/placebo was applied or not. Morelli et al. (2009) can be divided based on AVP or Terlipressin (TP) (Table 2).

**TABLE 2:** Basic characteristics, participants, intervention and outcomes.

Study	Study design	Cases (V/N)	Age (median, y)	CRF	VRA type	Dose for VRA	Dose for NE	Reported outcomes
Russell et al. (2008)	RCT	779 (397/382)	61.8	88 (11.3%)	Vasopressin	0.01–0.03 U/min	5–15 μg/min	RDFD, RFFD
Gordon et al. (2016) (hydrocortisone)	RCT	202 (101/101)	66	14 (6.9%)	Vasopressin	Titrated up to 0.06 U/min	Titrated up to 12 μg/min	RF rate, RFFD, RRT-use rate
Gordon et al. (2016) (placebo)	RCT	207 (104/103)	66	13 (7%)	Vasopressin	Titrated up to 0.06 U/min	Titrated up to 12 μg/min	RF rate, RFFD, RRT-use rate
Hajjar et al. (2019)	RCT	250 (125/125)	64	NR	Vasopressin	0.01–0.06 U/min	10–60 μg/min	AKI rate, RRT-use rate, RFFD
Mehta et al. (2013)	RCT	121 (65/56)	63.9	26 (21.5%)	Vasopressin	0.01–0.03 U/min	5–15 μg/min	Serum creatinine level
Gordon et al. (2010)	RCT	779 (397/382)	61.8	88 (11.3%)	Vasopressin	0.01–0.03 U/min	5–15 μg/min	AKI rate, RRT-use rate, RF rate, Serum creatinine level
Hammond et al. (2019)	Retrospective cohort	96 (48/48)	58.6	19 (20%)	Vasopressin	0.04 U/min	23.5 μg/min	RRT-use rate
Hammond et al. (2018)	Retrospective cohort	82 (41/41)	52	44 (50%)	Vasopressin	0.04 U/min	5–22 ug/min	RRT-use rate
Morelli et al. (2009) (TERLIVAP) −1 (TP)	RCT	30 (15/15)	67	0 (0%)	Terlipressin	1.3 μg/kg/hour	15 μg/min	RRT-use rate, Urinary output, Serum creatinine level
Morelli et al. (2009) (TERLIVAP) −2 (AVP)	RCT	30 (15/15)	66	0 (0%)	Vasopressin	0.03 U/min	15 μg/min	RRT-use rate, Urinary output, Serum creatinine level
Choudhury et al. (2017)	RCT	84 (42/42)	46.76	NR	Terlipressin	1.3–5.2 μg/min	7.5 to 60 ug/min	AKI rate
Dünser et al. (2003)	RCT	48 (24/24)	68	NR	Vasopressin	4 U/h	2.26 μg/kg/min	Serum creatinine level
.Patel et al. (2002)	RCT	24 (13/11)	68	NR	Vasopressin	0.06 U/min	17 μg/min	Serum creatinine clearance, Urinary output
Lauzier et al. (2006)	RCT	23 (13/10)	51	NR	Vasopressin	0.04–0.2 U/min	0.1–2.8 μg/kg/min	Serum creatinine clearance, Urinary output
Hall et al. (2004)	Retrospective cohort	99 (50/49)	67.1	14 (14.2%)	Vasopressin	0.04 U/min	1 μg/kg/min	Serum creatinine level, Urinary output, RFFD, AKI rate, RRT-use rate

Abbreviation: AKI, acute kidney injury; CRF, chronic renal failure; VRA, vasopressin receptor agonists; VAP, vasopressin; NE, norepinephrine; RDFD, Renal dysfunction-free days; RFFD, Renal failure-free days; RF, renal failure; RRT, renal replacement therapy; NR, not reported.

### 3.3 Quality and bias assessment

**FIGURE 2:**
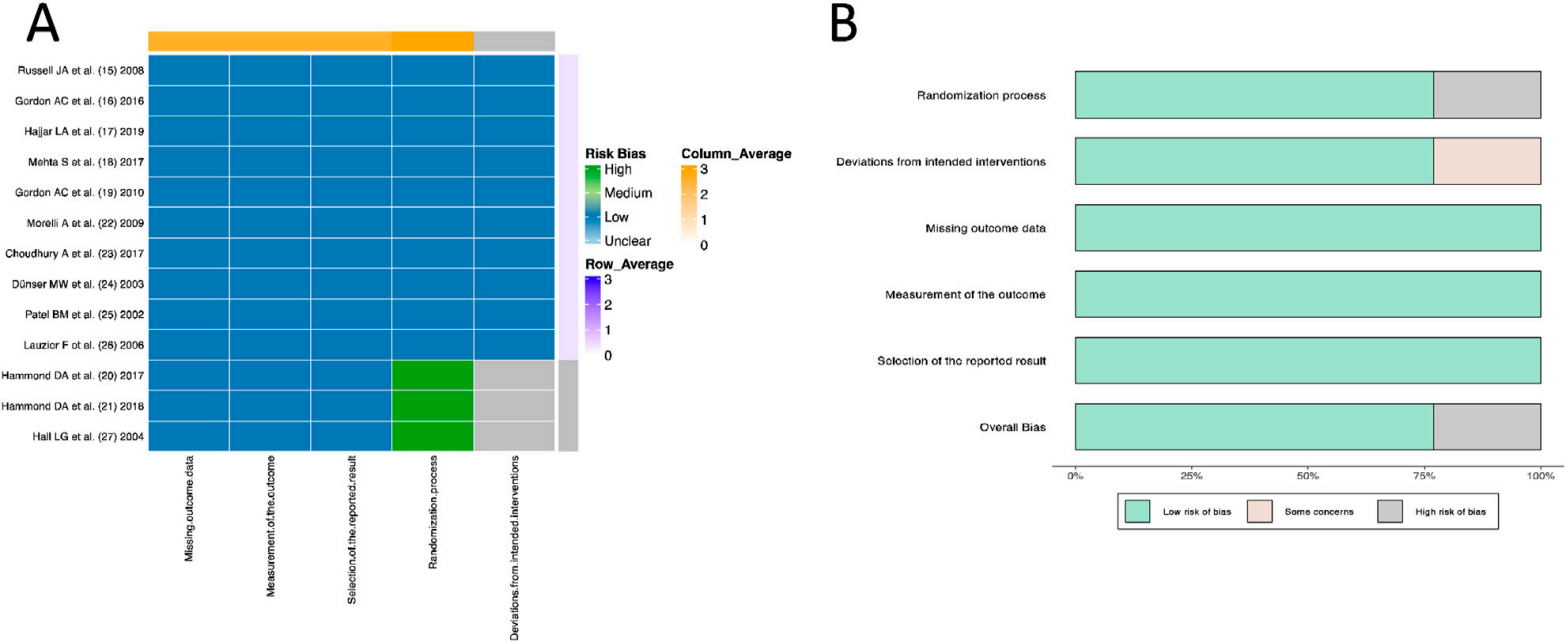
Quality evaluation and bias assessment based on Cochrane Risk of Bias V2.0. **(A)** Heatmap. **(B)** Histogram.

All 10 RCT studies (Russell et al., 2008; Gordon et al., 2016; Hajjar et al., 2019; Mehta et al., 2013; Gordon et al., 2010; Morelli et al., 2009; Choudhury et al., 2017; Dünser et al., 2003; Patel et al., 2002; Lauzier et al., 2006) provided detailed descriptions of the randomization process and blinding methods, with complete dropout case records and were therefore rated as “low risk.” However, the three retrospective cohort studies (Hammond et al., 2019; Hammond et al., 2018; Hall et al., 2004) had a “high risk” of randomization and “some concerns of risk” in terms of “deviations from intended interventions” due to the lack of randomization process and blinding methods (Figure 2).

### 3.4 Evaluation of long-term renal outcomes

#### 3.4.1 AKI rate

**FIGURE 3:**
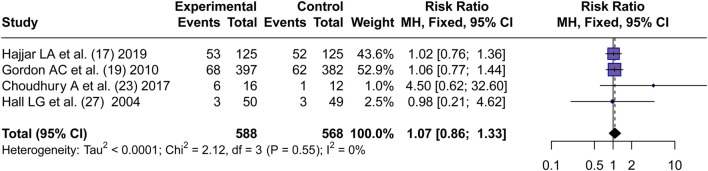
Forest plot of pooled RRs of AKI rates. “Experimental” represents “vasopressin,” while “control” represents “norepinephrine.” RR, risk ratio.

Four articles (Hajjar et al., 2019; Gordon et al., 2010; Choudhury et al., 2017; Hall et al., 2004) reported the incidence of AKI. By using a fixed effects model, the RR values of four articles were pooled. No significant difference in the incidence of AKI between AVP and NE in septic shock was found (RR = 1.07, 95% CI [0.86; 1.33], P = 0.53) (Figure 3).

#### 3.4.2 Days free of renal failure

**FIGURE 4:**
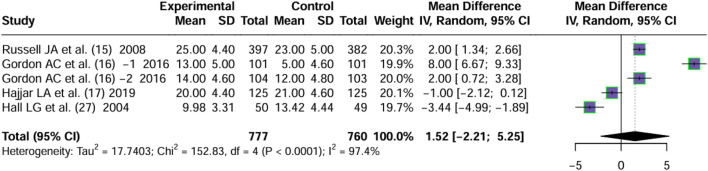
Forest plot of pooled MDs of days free of RF. “Experimental” represents “vasopressin,” while “control” represents “norepinephrine.” RF, renal failure; MD, mean difference.

Four articles (Russell et al., 2008; Gordon et al., 2016; Hajjar et al., 2019; Hall et al., 2004) reported on days free of renal failure. Significant heterogeneity was observed between the trials (*I* ^2^ = 97.4%, P < 0.0001). By using a random effects model, the MD values of four articles were pooled. No significant difference in the days free of renal failure of patients treated with vasopressin and norepinephrine in septic shock was observed (MD = 1.52, 95% CI [−2.21; 5.25], P = 0.43) (Figure 4).

#### 3.4.3 Renal failure rate

**FIGURE 5:**
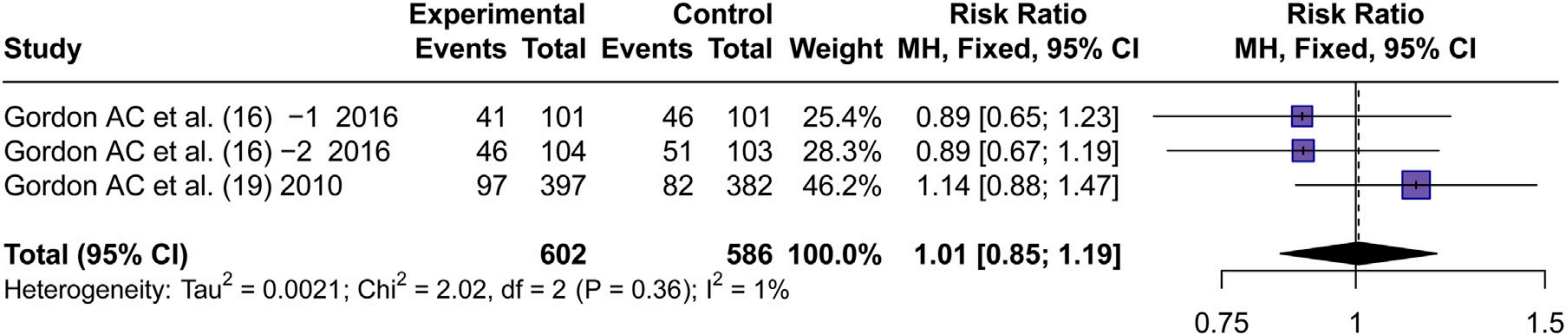
Forest plot of pooled RRs of RF rate. “Experimental” represents “vasopressin,” while “control” represents “norepinephrine.” RF, renal failure; RR, risk ratio.

Two articles (Gordon et al., 2016; Gordon et al., 2010) reported the incidence of Renal failure. By using a fixed effects model, the RR values were pooled. No significant difference in the incidence of RF between AVP and NE was observed (RR = 1.01, 95% CI [0.85; 1.19], P = 0.94) (Figure 5).

#### 3.4.4 RRT-use rate

**FIGURE 6:**
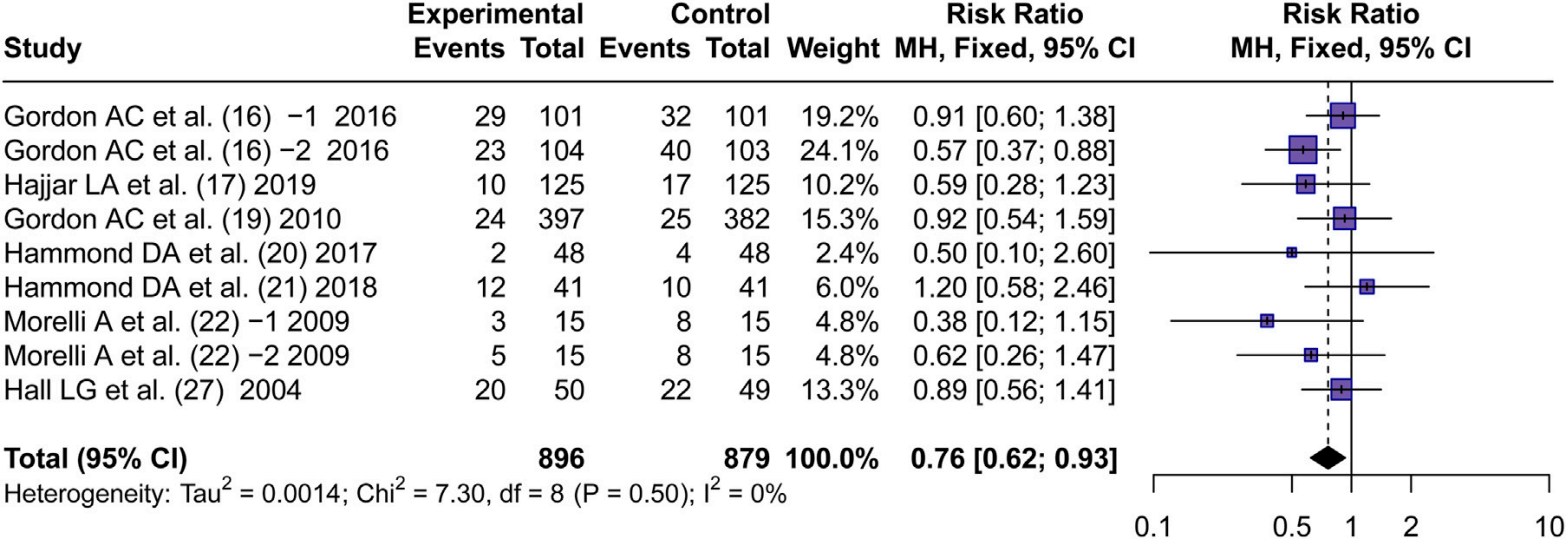
Forest plot of pooled RRs of RRT-use rate. “Experimental” represents “vasopressin,” while “control” represents “norepinephrine.” RRT, renal replacement therapy; RR, risk ratio.

Seven articles (Gordon et al., 2016; Hajjar et al., 2019; Gordon et al., 2010; Hammond et al., 2019; Hammond et al., 2018; Morelli et al., 2009; Hall et al., 2004) reported RRT use rate. By using a fixed effects model, the RR values of 7 articles were pooled. It was found that there was a significant difference in RRT-use rate between AVP and NE groups (RR = 0.76, 95% CI [0.62; 0.93], P < 0.01) (Figure 6). This corresponds to an approximate absolute risk reduction of 5%–10%, suggesting a potentially meaningful clinical effect.

### 3.5 Evaluation of short-term renal outcomes

#### 3.5.1 Serum creatinine level

**FIGURE 7:**
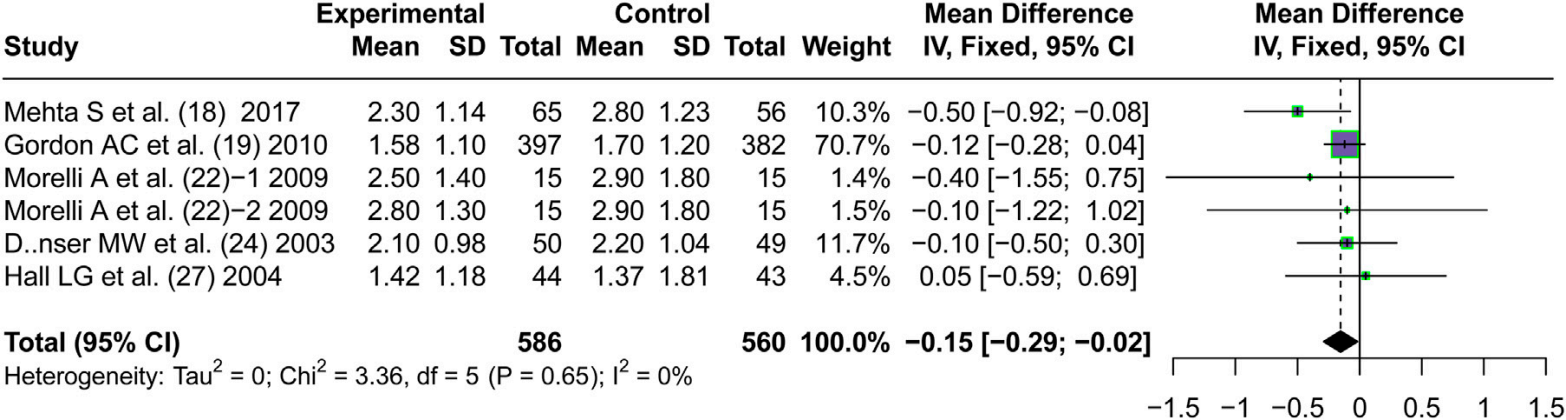
Forest plot of pooled MDs of serum creatinine level. “Experimental” represents “vasopressin,” while “control” represents “norepinephrine.” MD, mean difference.

Five articles (Mehta et al., 2013; Gordon et al., 2010; Morelli et al., 2009; Dünser et al., 2003; Hall et al., 2004) reported the short-term serum creatinine levels after the application of vasopressors. A lower short-term serum creatinine level was observed on patients treated with AVP than those treated with NE (MD = −0.15, 95% CI [−0.29; −0.02], P = 0.028) (mg/dL) (Figure 7). Although this difference was statistically significant, it did not exceed the reported minimal clinically important difference (MCID) for serum creatinine (approximately 0.3 mg/dL) defined by KDIGO guidelines (Khwaja, 2012), suggesting that the reduction may have limited clinical relevance despite statistical significance.

#### 3.5.2 Creatinine clearance

Only two articles (Patel et al., 2002; Lauzier et al., 2006) reported on creatinine clearance. Studies (Patel et al., 2002) reported that after 4 h of infusion, the creatinine clearance rate of patients receiving AVP treatment was significantly higher than that of NE. Stuides (Lauzier et al., 2006) reported that the creatinine clearance rate of patients receiving AVP was higher than that of NE at 12, 24, and 48 h after treatment, with statistical significance at 24 h.

#### 3.5.3 Urine output

**FIGURE 8:**
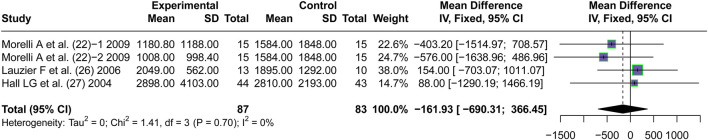
Forest plot of pooled MDs of urine output. “Experimental” represents “vasopressin,” while “control” represents “norepinephrine.” MD, mean difference.

Three articles (Morelli et al., 2009; Lauzier et al., 2006; Hall et al., 2004) reported the short-term urinary output after the application of vasopressors. No significant difference in short-term urine output between patients treated with AVP and NE in Septic shock was observed (MD = −161.93, 95% CI [−690.31; 366.45], P = 0.55) (Figure 8).

### 3.6 Subgroup analysis

**FIGURE 9:**
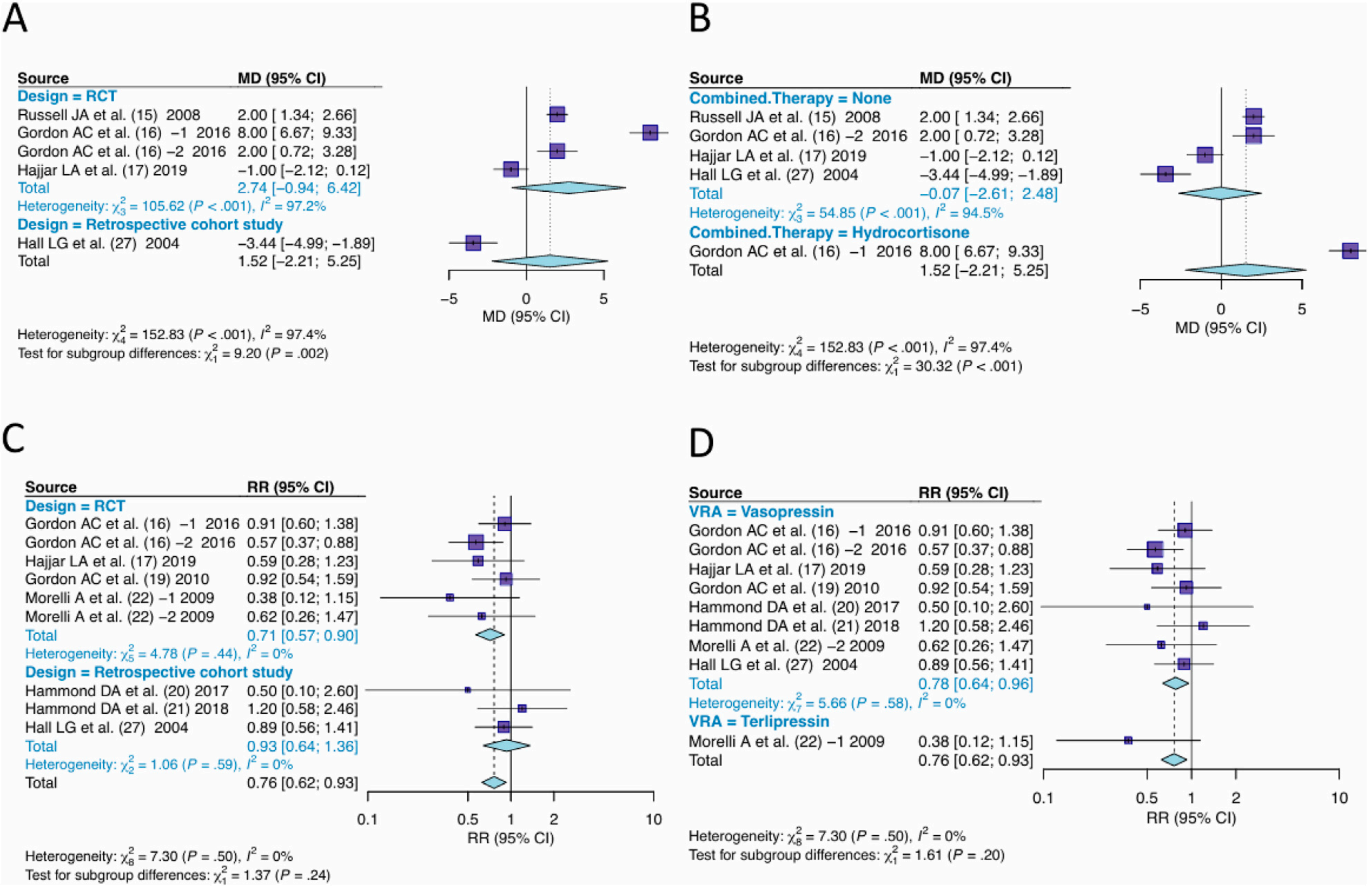
Subgroups analysis. **(A)** Studies were grouped by design for outcome of days free of renal failure. **(B)** Studies were grouped by the combined therapy for outcome of days free of renal failure. **(C)** Studies were grouped by design for outcome of RRT-use rate. **(D)** Studies were grouped by the type of VRA for outcome of RRT-use rate. VRA, Vasopressin receptor agonists; RRT, Renal replacement Therapy.

In the pooling of effect sizes for days free of renal failure, significant heterogeneity was observed. The studies were sub-grouped for investigation of the sources of heterogeneity by “design” (Figure 9A) or “the combined therapy” (Figure 9B). Significant differences were observed between subgroups by design (P = 0.002), or by the combined therapy (P < 0.001), indicating both “design” and “the combined therapy” were the sources of heterogeneity.

In the pooling of RRT-use rate effect sizes, no significant heterogeneity was observed. The studies were still sub-grouped based on “design” and “type of VRA” for subgroup analysis. The difference between subgroups by design was not significant (P = 0.24) (Figure 9C), nor by type of VRA (P = 0.20 (Figure 9D), indicating no significant heterogeneity difference between RCT and Cohort studies, nor between Vasopressin and Terlipressin.

### 3.7 Influence analysis

**FIGURE 10:**
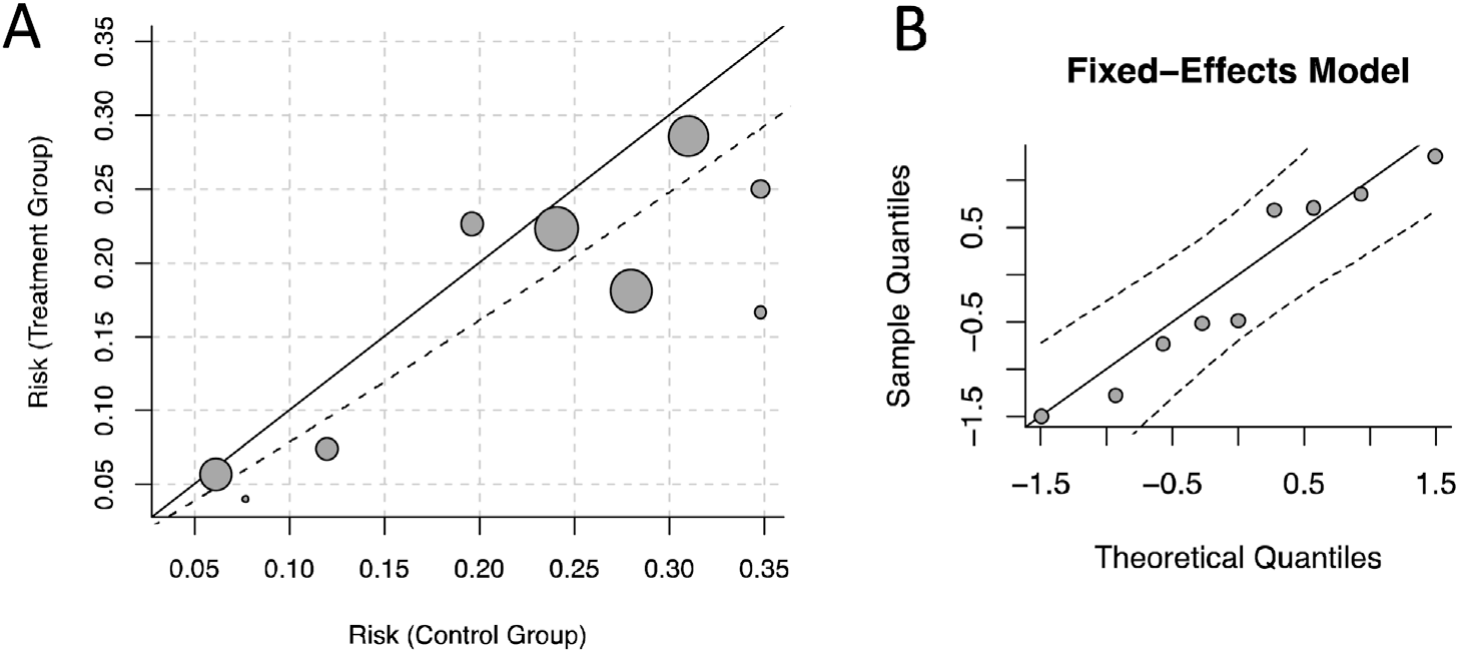
Influence analysis. **(A)** Labbe diagram. **(B)** Norm Q-Q Chart.

In the analysis of RRT-use rate, we used Labbe plot and norm QQ plot for influence analysis, and found that the study distribution was relatively concentrated, without obvious outlier, indicating that the results of the study was stable (Figure 10).

### 3.8 Publication bias

**FIGURE 11:**
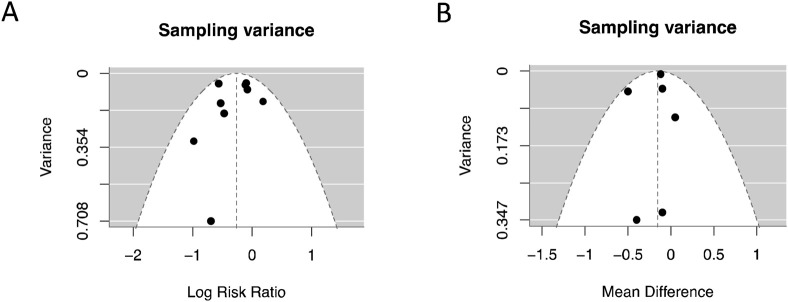
Publish bias analysis and funnel plot. **(A)** RRT-use rate; **(B)** Serum creatinine level.

In the analysis of publication bias for RRT-use rate and serum creativity level, we used a funnel plot to show the publication bias of the two outcome measures (Figure 11). The funnel plot did not show significant asymmetry on both sides, indicating minimal publication bias.

## 4 Discussion

Arginine vasopressin (AVP), or simply vasopressin, is a nonapeptide hormone composed of nine amino acids synthesized by neurons in the hypothalamus (Bankir et al., 2017). The impaired AVP secretion system leads to decreased serum AVP levels, which contributes to the loss of vascular tone and hemodynamic instability in patients with septic shock (Christ-Crain, 2019). Accordingly, the administration of exogenous AVP has been proposed as a therapeutic strategy to restore vascular tone and improve circulatory stability in septic shock. In the RCT conducted by Daley et al. (2013), the effects of AVP and NE monotherapy within the first 6 h of admission were compared in patients with septic shock. The study found that AVP was equally effective as NE in maintaining MAP above 65 mmHg. Similarly, a large RCT by Russell et al. (2008) investigated the effects of low-dose AVP versus NE and found no significant difference in 28- and 90-day mortality or in the incidence of cardiovascular adverse events. Interestingly, patients receiving AVP exhibited fewer Q waves on electrocardiography compared to those receiving NE, suggesting a potential cardioprotective effect of AVP.

Despite these findings, there remains uncertainty regarding whether AVP, while effectively maintaining MAP, can also improve perfusion of other key organs, especially in this article, the kidneys, and reduce the extent of organ dysfunction in septic shock. In the study by Okazaki et al. (2020), a sheep model of sepsis combined with AKI was established and randomly assigned to receive either AVP or norepinephrine (NE) (n = 7 per group). The findings indicated that AVP administration did not exacerbate medullary ischemia or hypoxia, nor did it reduce mesenteric blood flow, suggesting AVP may confer superior renal protection compared to NE. However, as this was an animal-based RCT and not a human study, it was excluded from the current meta-analysis.

This meta-analysis included 13 eligible studies, 10 RCTs and 3 retrospective cohort studies. All studies reported outcomes related to either short-term or long-term renal function. Notably, the studies by Russell et al. (2008) and Gordon et al. (2010) were both derived from the Vasopressin and Septic Shock Trial (VASST) but reported distinct renal outcome measures. The RCT conducted by Hajjar et al. (2019) randomized 250 patients to receive either AVP or NE to investigate the outcomes included 90-day mortality, the incidence of adverse events, the incidence of AKI, rate of renal replacement therapy (RRT) use, and renal failure–free days (RFFD). A cohort study by Hammond et al. (2019) that included in this meta-analysis compared long-term outcomes of early intervention with AVP versus NE in patients with septic shock and reported outcomes included 28-day mortality, hospital length of stay, and RRT utilization. These studies provided important data for assessing the renal effects of AVP compared to NE in clinical settings.

The pooled results suggest that AVP, including its derivative Terlipressin (TP), is associated with a significant short-term reduction in serum creatinine levels compared to NE. The statistically significant reduction in serum creatinine (−0.15 mg/dL) did not reach the minimal clinically important difference threshold (≈0.3 mg/dL), indicating that the improvement may not translate into meaningful clinical benefit. However, it indeed brings us with truly benefit and alternative management for septic shock. Furthermore, creatinine clearance was observed higher in the AVP group, while no significant difference in urine output. In terms of long-term outcomes, the use of RRT was significantly lower among patients treated with AVP. However, no significant differences were observed between AVP and NE in the incidence of AKI, RF rate, or the days free of renal failure. These findings indicate that while AVP may not significantly reduce the overall incidence of kidney injury or prevent renal failure, it demonstrates renal protective effects during its administration. Specifically, AVP appears to enhance short-term renal function by improving creatinine clearance and lowering serum creatinine levels, and it may reduce the need for RRT in the long term.

A single-case study (Holmes et al., 2001) reported that administration of AVP significantly increased urine output in a patient with septic shock. However, pooled data from three studies (Morelli et al., 2009; Lauzier et al., 2006; Hall et al., 2004) exhibited no difference in early urine output between the two intervention groups. Regarding creatinine clearance, Patel et al. (2002) observed that after 4 h of infusion, the mean creatinine clearance in the AVP group was significantly higher (23 vs. 12.5 mL/min). Similarly, Lauzier et al. (2006) found that creatinine clearance was higher at 12, 24, and 48 h post-treatment in the AVP group, with values reaching an average of 122 mL/min at 24 h, compared to 54 mL/min in the NE group. These findings suggest that AVP may exert a renal protective effect, potentially mitigating the nephrotoxic effects of NE, such as reductions in glomerular filtration rate, creatinine clearance, and urine output.

Renal injury is a frequent and serious complication of septic shock and commonly observed in patients with septic shock when admission. In this meta-analysis, most RCTs include patients with co-existing chronic kidney disease (CKD), with prevalence ranging from 6.9% to 50%. Therefore, in septic shock patients with concurrent acute and chronic renal dysfunction, AVP may represent a more favorable alternative to NE as a vasopressor, offering potential renal benefits while maintaining hemodynamic stability.

The pooled effect size of RRT-use rate was significantly lower in the AVP group, supporting the potential renal benefits of AVP. This finding aligns with Al-Husinat et al. (2023). Although an established MCID is unavailable for binary outcomes of RRT use rate, the observed 24% relative risk reduction is likely to be clinically relevant, particularly in patients with moderate to high baseline risk.

However, we found no significant differences in terms of AKI incidence, RF rate, or the number of renal failure–free days. We speculated that this may be related to the inclusion of too few studies and samples in the data pooling of these outcomes. Additionally, the development of renal injury or failure in septic shock is likely influenced more by the underlying disease progression than by the choice of vasopressor alone.

In this meta-analysis, the AVP doses for all studies were controlled between 0.01 and 0.06 U/min. Klinzing et al. (2003) investigated the therapeutic effect of high-dose AVP 0.47 (0.06–1.8) IU/min and found that high-dose AVP can significantly reduce cardiovascular output while maintaining liver blood flow perfusion, but it is not conducive to the distribution of intestinal blood flow and has adverse effects on intestinal mucosa. Therefore, high-dose AVP cannot replace NE. Animal experiment (Malay et al., 2004) had shown that medium and high-dose AVP doses can cause very heterogeneous vasoconstrictive effects on different organs. An increase in vasopressin dose can improve cerebral perfusion but will significantly reduce mesenteric and renal blood flow. In fact, in the treatment practice of septic shock, the safe dose range of exogenous AVP is very narrow, usually between 0.01 and 0.06 U/min (Fage et al., 2023). This also reminds healthcare givers to pay special attention to possible intestinal mucosal lesions in patients during the application of AVP.

Gordon et al. (2016) conducted a study involving 409 patients with septic shock, dividing them into two sub-studies (comprising four randomized groups) to evaluate the potential interaction between AVP and hydrocortisone (HCT). No mortality difference was found in between AVP+HCT/AVP+Placebo groups. However, significantly shorter durations of hospital and ICU stay suggests that the combination therapy may offer clinical benefits beyond survival. Similarly, study by Torgersen et al. (2011) included 159 patients, of whom 76 received combined AVP+HCT therapy and observed a lower mortality. Studies involved the combination of AVP and HCT brings heterogeneity in this meta-analysis, the findings suggest that the combination of AVP and HCT may confer additional benefits to patients with septic shock. These benefits may arise from potential synergistic effects on vascular responsiveness, immune modulation, and other physiological pathways.

In this study, only two trials involving terlipressin (TP) were included. Subgroup analysis revealed no significant difference in AVP and TP subgroups. In the TERLIVAP trial (Morelli et al., 2009), TP was associated with reduced catecholamine requirements and a lower incidence of rebound hypotension over the other two vasopressors. Given that only two TP-related studies were included, the strength of the evidence regarding its potential renal protective effects remains limited. Further RCTs are needed to clarify the role of TP in septic shock management and its comparative efficacy with AVP.

In this meta-analysis, all RCTs included were high-quality studies, while there may be some bias introduced by the control process of the 3 cohort studies. Subgroup analysis found that the study design contributes to the heterogeneity of the days free of RF, whereas it did not significantly contribute to the overall heterogeneity of RRT-use rate. The Labbe and norm QQ plots indicate that there were no significant outliers in this study for the outcome of RRT-use rate, suggesting that the results were stable and reliable.

However, several limitations should be acknowledged. First, some key renal function outcomes such as AKI incidence, RF rate, and Days free of renal failure, were derived from studies with small sample sizes and low quality of evidence. Second, some important renal function outcomes such as urinary albumin level, incidence of chronic kidney disease (CHD) were not analyzed in this meta-analysis due to a lack of available data, Third, this study was restricted to adult patients, as the dosage and sensitivity of NE and AVP in children are different from those in adults, and inclusion of pediatric population could have introduced significant heterogeneity. Thus, further in-depth exploration and analysis are still needed for research on this topic.

## 5 Conclusion

Vasopressin, as a second-line treatment of patients with septic shock, is associated with a significant short-term reduction in serum creatinine levels and a decreased long-term need for renal replacement therapy compared to norepinephrine. The application of vasopressin in the management of septic shock has greater potential for kidney protection. However, its use does not show a significant association with early urine output, the incidence of acute kidney injury, or the occurrence of renal failure. Given the small sample size and the low quality of evidence for these outcomes, additional high-quality studies are needed to further validate these findings.
